# Efficacy of Upadacitinib in Treating Alopecia Areata, Atopic Dermatitis, and Th1 Comorbidities in Pediatric Patients: A Comprehensive Case Series and Literature Review

**DOI:** 10.3390/jcm14113881

**Published:** 2025-05-30

**Authors:** Chiara Battilotti, Giulia Azzella, Annunziata Dattola, Alfredo Rossi, Francesca Svara, Camilla Chello, Ester Del Duca, Giovanni Paolino, Steven P. Nisticò, Giovanni Pellacani, Teresa Grieco

**Affiliations:** 1 Dermatologic Clinic, Sapienza University of Rome, 00185 Rome, Italy; chiara.battilotti@gmail.com (C.B.); azzellagiulia1996@gmail.com (G.A.); annunziata.dattola@uniroma1.it (A.D.); alfredo.rossi@uniroma1.it (A.R.); camilla.chello@uniroma1.it (C.C.); ester.delduca@gmail.com (E.D.D.); steven.nistico@uniroma1.it (S.P.N.); giovanni.pellacani@uniroma1.it (G.P.); teresa.grieco@uniroma1.it (T.G.); 2 Unit of Dermatology, IRCCS Ospedale San Raffaele, 20132 Milan, Italy; paolino.giovanni@hsr.it; 3 Unit of Clinical Dermatology, Università Vita-Salute San Raffaele, 20132 Milan, Italy

**Keywords:** atopic dermatitis, alopecia areata, upadacitinib, JAK inhibitors, pediatric dermatology

## Abstract

Alopecia areata (AA) and atopic dermatitis (AD) are complex immune-mediated conditions that frequently coexist in pediatric patients, complicating treatment approaches. Upadacitinib, a selective JAK1 inhibitor, modulates both Th1 and Th2 pathways and is approved for AD in adolescents and adults. This study presents a case series of three adolescent patients with refractory AA and AD treated with upadacitinib 15 mg/day for 12 months, alongside a comprehensive literature review. All patients demonstrated rapid remission of AD symptoms within the first month and progressive hair regrowth, with SALT scores significantly improving at six and twelve months. No severe adverse events were reported. Notably, one patient achieved complete regrowth despite the presence of ophiasis, a pattern typically associated with poor prognosis. Our literature review identified only four previous pediatric cases successfully treated with upadacitinib, highlighting the novelty of our findings. These cases, together with our experience, suggest that upadacitinib offers a safe and effective therapeutic option for pediatric patients with concomitant AA and AD, including those who failed conventional or biologic therapies such as dupilumab. Larger, controlled studies are needed to confirm long-term efficacy and safety. Our results also support the potential role of upadacitinib in managing multiple Th1/Th2-mediated comorbidities in pediatric populations.

## 1. Introduction

Alopecia areata (AA) is an autoimmune disorder characterized by non-scarring hair loss, which can range from small patches to complete hair loss across the scalp and body [1,2]. It is primarily mediated by T-helper cell type 1 (Th1) [3]. Atopic dermatitis (AD) is a chronic inflammatory skin condition primarily driven by T-helper cell type 2 (Th2) pathways [4]. The pathogenesis of AD is highly complex. In the acute phase, the alarmins IL-25, IL-33, and Thymic Stromal Lymphopoietin (TSLP), released by epidermal keratinocytes, primarily switch naïve dermal and lymph node T cells to Th2 lymphocytes via the OX40 ligand [5]. Recent studies have shown that Th2-, IL4-, IL13-, and IL5-related cytokines in chronic AD coexist with other cytokine pathways, such as Th1, Th17, and Th22 [6]. IL-4, IL-5, IL-13, and IL-31 play a central role in promoting B-cell class switching to IgE, activating eosinophils, and contributing to skin barrier dysfunction and pruritus. IL-17A and IL-22 play a significant role in epidermal thickening and barrier dysfunction by promoting keratinocyte proliferation in the chronic phase of AD [7]. Moreover, Th1 cytokines, including IFN-γ and TNF-α, become relevant in perpetuating inflammation and immune responses [5]. This multifaceted cytokine milieu could be related to low responsiveness and therapy failure, particularly with Th2 biologic drugs such as dupilumab and tralokinumab. It also notes that patients affected by atopic conditions can develop autoimmune diseases (AIDs), such as AA, Crohn’s disease, and rheumatoid arthritis, over their lifetime [8]. Understanding these complex immune mechanisms is crucial for developing targeted therapies to effectively manage AD and Th1 comorbidities in the same patients. Traditional treatments for pediatric AD and AA, such as topical corticosteroids, calcineurin inhibitors, and systemic immunosuppressants (such as cyclosporine), often have limited long-term efficacy and notable side effects [9,10]. Dupilumab, approved for moderate-to-severe AD in children aged 6 months and older, offers a safer and more effective alternative [11]. While not approved for AA, some studies suggest it may benefit patients with both AD and AA [12]. However, its efficacy in AA alone remains uncertain, highlighting the need for new targeted therapies to improve outcomes in these patients. Janus Kinases (JAKs) are a family of transmembrane cellular proteins that mediate cytokines and growth factor signals from specific receptors of the cell surface to the cellular nucleus. JAKs are represented by four distinct molecules: JAK1, JAK2, and Tyk2 are found in a wide range of tissues, while JAK3 is primarily present in hematopoietic cells. The activation of the JAK-STAT signaling pathway regulates the expression of genes mediating numerous biological processes, including inflammation, immune responses, and hematopoiesis [13]. By inhibiting JAKs, JAK inhibitors (JAKi) can block the signaling of several pro-inflammatory cytokines, such as IL-4, IL-5, and IL-13 (Th2 cytokines), as well as IFN-γ (a Th1 cytokine). This broad mechanism of action allows JAKi to modulate both Th1 and Th2 pathways, making them effective in treating a variety of inflammatory and autoimmune diseases, as observed in AA and AD [14]. Upadacitinib is an oral, second-generation JAK1 inhibitor currently approved by the FDA and EMA for the treatment of rheumatoid arthritis, axial spondylarthritis, Crohn’s disease (CD), ulcerative colitis, and atopic dermatitis in adult patients. It is also approved for adolescents aged 12 years and older with AD [15]. Recent evidence proposes upadacitinib as a promising option for treating AD coexisting with inflammatory bowel diseases (IBDs) or other Th1 autoimmune conditions, such as AA, although it is not yet approved for this specific indication [16]. This report discusses three/adolescent cases that were simultaneously affected by AD and Th1 comorbidities—AA and CD, observed in one of our cases. We treated our patients with upadacitinib, exploring the efficacy and long-term safety of this therapeutic approach.

## 2. Materials and Methods

### 2.1. Study Design and Patients

This retrospective single-center study involved adolescent patients with AD who also had coexisting AA and were treated with upadacitinib between March 2023 and July 2024 at the Dermatology Department of Umberto I Hospital, Sapienza University of Rome, Italy. The diagnoses of AA and AD were clinically confirmed by dermatologists (T.G. and C.C.) based on their respective clinical features. The criteria for inclusion were (a) patients aged between 12 and 17 years with a confirmed diagnosis of AA and AD; (b) patients with AA who had not responded to previous topical and systemic treatments; and (c) patients treated with upadacitinib for at least 12 months. Exclusion criteria were (a) adult patients and (b) patients with a contraindication to treatment with upadacitinib.

### 2.2. Therapeutic Regimen

Upadacitinib was administered at a daily dose of 15 mg, in line with the recommended dosage for adolescents with AD [17]. Throughout the study period, no additional therapies, either topical or systemic, were given to the patients for the management of AA or AD.

### 2.3. Data Collection and Outcomes

Effectiveness outcomes, including the Severity of Alopecia Tool (SALT) score, Eyebrow Assessment (EBA) score, Eyelash Assessment (ELA), Eczema Area and Severity Index (EASI) score, Itch Numerical Rating Scale (Itch NRS), Sleep Numerical Rating Scale (Sleep NRS), Investigator’s Global Assessment (IGA) score, and Dermatology Life Quality Index (DLQI) score, were recorded at baseline and at each follow-up visit. Safety was assessed through the systematic monitoring of adverse events (AEs) at each visit, accompanied by regular assessments of laboratory values to detect any significant changes (Supplementary Materials).

### 2.4. Literature Review

A comprehensive literature search of PubMed and Scopus was conducted throughout July 2024. The search strategy was conducted without restrictions on language or year of publication. Search terms included ‘alopecia areata’, ‘ophiasis’, ‘alopecia universalis’, ‘alopecia totalis’, ‘hair loss’, ‘upadacitinib’, ‘children’, ‘adolescents’, and ‘pediatric’. Boolean operators AND and OR were employed to combine the search terms as follows: (alopecia areata OR ophiasis OR alopecia universalis OR alopecia totalis OR hair loss) AND (upadacitinib) AND (children OR adolescents OR pediatric). Inclusion criteria were as follows: articles involving pediatric or adolescent patients (defined as individuals under 18 years of age) with AA who were treated with upadacitinib. Eligible studies included case reports, clinical trials, and observational studies that provided detailed clinical outcomes. Exclusion criteria included studies focusing on adult patients (aged 18 years or older) or those that did not specifically address the treatment of alopecia areata with upadacitinib. Additionally, articles lacking primary patient data, such as reviews without clinical case details, were excluded.

## 3. Results

Of the four pediatric patients with concurrent AA and AD who were being treated with upadacitinib, three met the inclusion criteria. Individual cases are described below. Table 1 and Table 2 summarize the characteristics of the patients and the duration and outcome of the therapy, respectively. Table 3 outlines the laboratory protocols performed at baseline and at each follow-up visit.

### 3.1. Case 1

**Figure 1:**
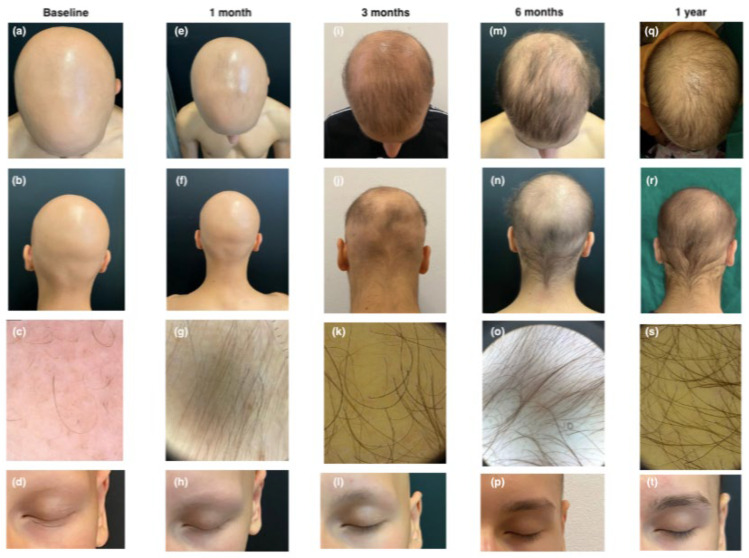
Clinical and trichoscopic presentation at baseline (**a**–**d**); after 1 month of therapy with upadacitinib (**e**–**h**); after 3 months of therapy with upadacitinib (**i**–**l**); after 6 months of therapy with upadacitinib (**m**–**p**); and after 12 months of therapy with upadacitinib (**q**–**t**).

The first patient was a 13-year-old boy with a history of moderate-to-severe early-onset AD and coexistent universal AA since 2022, along with allergic rhinoconjunctivitis. The patient was previously treated with dupilumab (200 mg bi-monthly) for two years, which resulted in only partial improvement of AD symptoms. At baseline, the patient had a SALT score of 100 (total scalp hair loss) and significant loss of eyebrows and eyelashes (EBA and ELA scores of 0). The patient was also scored for AD, resulting in EASI 10, indicating moderate disease with skin fold involvement. IGA score was ≤1, Itch NRS was 6, and Sleep NRS was 4, reflecting the impact on sleep. Trichoscopy revealed active AA features, and AD scoring indicated moderate disease activity with a DLQI score of 10. To achieve AD Minimal Disease Activity (MDA), the therapy was switched to upadacitinib, 15 mg/day, in October 2023. After one month, partial regrowth of eyebrow and eyelashes was observed (EBA and ELA scores of 1), and the SALT score was 95. Trichoscopy revealed sporadic regrowth hairs and vellus hairs. Complete remission of AD symptoms (EASI, itch NRS, Sleep NRS, IGA, and DLQI all scored 0) was achieved. Three months later, we obtained significant improvement with substantial regrowth of eyelashes (EBA 2), eyebrows (ELA 2), and scalp hair, resulting in a SALT score of 35.3. Trichoscopy showed numerous terminal and thin regrowing hairs, with some residual yellow dots. The patient expressed satisfaction with the treatment outcomes. After six months, the child showed improvement, with a SALT score dropping to 21.8 and complete regrowth of eyebrows and eyelashes (EBA and ELA scores of 3). Trichoscopy revealed normal terminal hairs, and the patient maintained complete remission of AD, achieving MDA as well. At the twelve-month follow-up visit, further hair growth was observed with a SALT score of 14 (see Figure 1).

### 3.2. Case 2

**Figure 2:**
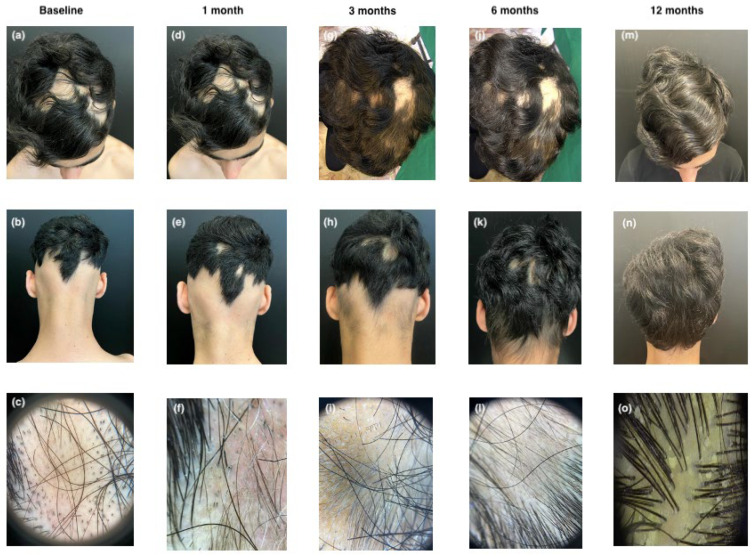
Clinical and trichoscopic presentation at baseline (**a**–**c**); after 1 month of therapy with upadacitinib (**d**–**f**); after 3 months of therapy with upadacitinib (**g**–**i**); after 6 months of therapy with upadacitinib (**j**–**l**); and after 12 months of therapy with upadacitinib (**m**–**o**).

The second patient, a 12-year-old boy, has had moderate AD since early childhood, coexisting with recently onset AA. After the failure of various topical and systemic traditional treatments, including corticosteroids and cyclosporine, which only partially controlled his AD, he was referred to our clinic. At baseline, the patient presented with ophiasis, a pattern of hair loss affecting the occipital and temporal scalp, and had a SALT score of 51.5. Trichoscopic examination revealed yellow dots, black dots, and exclamation mark hairs. The AD assessment showed an EASI score of 9, an IGA score of 1, a Sleep NRS of 4, an itch NRS of 6, and a DLQI of 8, indicating moderate disease activity. Treatment with upadacitinib began in March 2023 at a dose of 15 mg/day, following enrollment and baseline laboratory assessments. After one month, there was no significant clinical improvement in scalp hair, with a SALT score of 47.8. Trichoscopy still showed numerous black dots, but hypopigmented vellus hairs, circle hairs, and regrowing hairs were also observed. The patient’s AD symptoms were completely controlled. After three and six months, the patient’s SALT score improved to 35.3 and 15, respectively, with trichoscopy showing numerous regrowing and terminal hairs. At eleven months, nearly complete hair regrowth was observed, with a SALT score of 1.8. Trichoscopy revealed terminal hairs, with only a small patch of AA persisting in the left temporal area (see Figure 2).

### 3.3. Case 3

**Figure 3:**
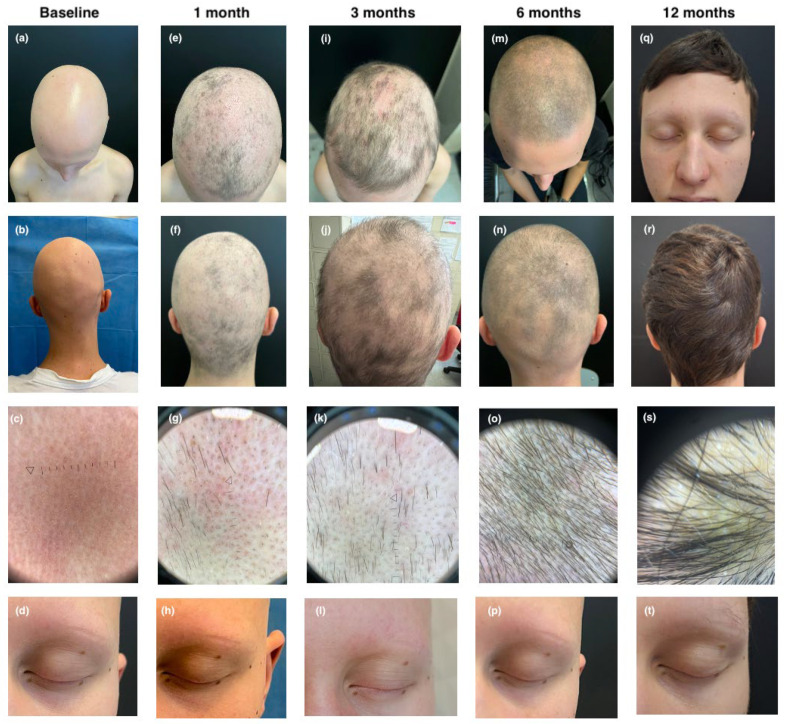
Clinical and trichoscopic presentation at baseline (**a**–**d**); after 1 month of therapy with upadacitinib (**e**–**h**); after 3 months of therapy with upadacitinib (**i**–**l**); after 6 months of therapy with upadacitinib (**m**–**p**); and after 12 months of therapy with upadacitinib (**q**–**t**).

The third patient, a 14-year-old male, had a history of CD, localized to the ileocolic region since 2020, and eosinophilic gastroenteritis involving the duodenum and ileum since 2021. Colonoscopy revealed hyperemic and edematous mucosa in the descending and sigmoid colon, accompanied by diffuse aphthous ulcerations. Esophagogastroduodenoscopy (EGDS) demonstrated mucosal hyperemia in the duodenum and ileum. In September 2022, he developed alopecia universalis, resulting in complete hair loss within three weeks. He also had early-onset AD from 18 months of age. Previous treatments, including oral methylprednisolone and dupilumab, had been unsuccessful, leading to his referral to our clinic. At baseline, the patient exhibited severe alopecia universalis with a SALT score of 100, indicating total hair loss, and a complete absence of eyebrows and eyelashes (EBA and ELA score of 0). His atopic dermatitis was active, with an EASI score of 8, an itch NRS of 6, a Sleep NRS of 5, an IGA of 2, and a DLQI of 10. Considering the coexisting conditions of CD, alopecia universalis, and AD, we decided to initiate treatment with a JAKi. Upadacitinib treatment commenced in March 2024 at a dosage of 15 mg/day, following initial enrollment and baseline laboratory evaluations. After one month of treatment, the patient demonstrated minimal clinical improvement, with a SALT score of 95 and EBA and ELA scores of 0, although sporadic hair regrowth was noted. AD symptoms were well controlled, with substantial improvement across all clinical indices, EASI, itch NRS, Sleep NRS, IGA, and DLQI, all of which decreased to 0. After three months, the patient’s alopecia showed marked improvement, with a SALT score of 78, indicating diffuse hair regrowth across the scalp. At the six-month follow-up visit, the patient showed marked improvement with a SALT score of 45 and minimal regrowth of eyebrows and eyelashes (EBA and ELA scores of 1). Further improvement was observed at the twelve-month follow-up, with a SALT score of 0 and ELA and EBA scores of 2 (see Figure 3). Follow-up gastrointestinal evaluation via colonoscopy and EGDS revealed significant clinical improvement. The EGDS showed no evidence of disease, while colonoscopy indicated only mild hyperemia of the sigmoid mucosa and near-complete resolution of aphthous ulcers.

### 3.4. SALT Score

**Figure 4:**
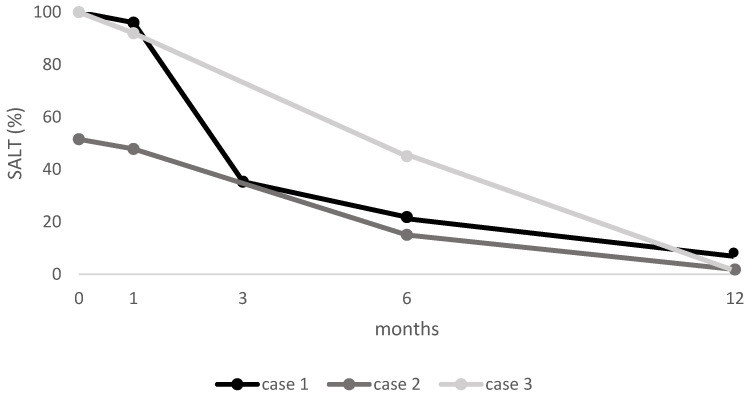
SALT score for each patient at baseline and at follow-up visits. SALT: Severity of Alopecia Tool.

At baseline, Cases 1 and 3 had a SALT score of 100%, while Case 2 had a SALT score of 51.5%. After one month, all cases showed initial improvement. By six months, patients exhibited substantial regrowth (SALT of 21.8, 15, and 45, respectively), with near-complete remission at 12 months (SALT of 14, 1.8, and 0, respectively) (see Figure 4).

### 3.5. EBA Score

**Figure 5:**
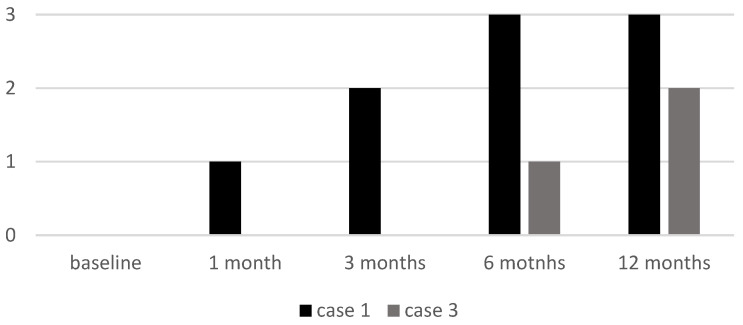
EBA score of two patients (Case 1 and Case 3) at baseline and at follow-up visits. EBA: Eyebrow Assessment.

Cases 1 and 3, both with alopecia universalis, had an initial EBA score of 0. Case 1 showed a progressive increase to 3 by six months, while Case 3 remained at 0 until six months, when it reached 1 with further improvement at the 12-month follow-up visit (EBA 2) (see Figure 5).

### 3.6. ELA Score

**Figure 6:**
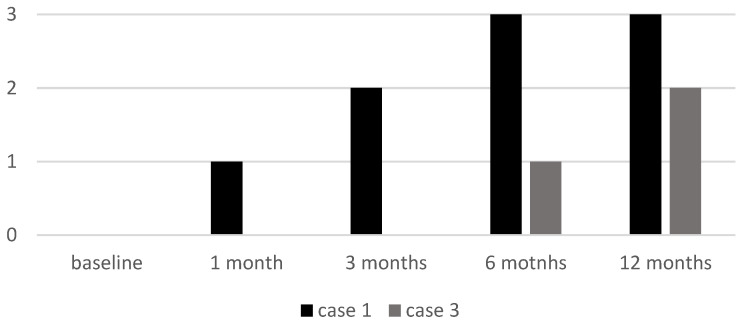
ELA score of two patients (Case 1 and Case 3) at baseline and at follow-up visits. ELA: Eyelash Assessment.

Cases 1 and 3, both with alopecia universalis, had an initial ELA score of 0. Case 1 showed a progressive increase to 3 by six months, while Case 3 remained at 0 until six months, when it reached 1, with further improvement at the 12-month follow-up visit (ELA 2) (see Figure 6).

### 3.7. Adverse Events

Throughout the treatment period, no adverse events were reported in any of the patients. Routine laboratory monitoring did not reveal any abnormalities, underscoring the safety profile of upadacitinib in these pediatric patients, except for a mild increase in CPK levels in patients 2 and 3, which returned to normal spontaneously within a few weeks.

### 3.8. Literature Review

The literature search in PubMed and Scopus yielded seven non-duplicated articles. After the screening process, four articles were deemed eligible, all of which were case reports. To date, only four adolescent/pediatric patients treated with upadacitinib have been documented in the literature. Table 4 summarizes the key clinical characteristics and the therapy outcomes with upadacitinib in these patients.

## 4. Discussion

### 4.1. Pathogenesis and Therapeutic Challenges in Pediatric AA and AD

AA is an autoimmune condition characterized by a cytotoxic T-cell response against hair follicles. Although the precise pathogenesis of AA remains unknown, it is believed to result from the loss of immune privilege on the anagen hair bulb, influenced by both genetic and environmental factors [22]. The management of AA in pediatric patients, particularly when it coexists with Th2-type conditions such as AD, remains a clinical challenge due to the absence of a specific drug for treating these categories. Therapeutic options are mainly limited to corticosteroids, irritants, topical sensitizers, and immunosuppressive agents [10]. Interestingly, approximately 25% of patients with AA are pediatric, and AA and AD coexist in about 16–33% of children and adolescents [23]. These two conditions exhibit multiple similarities in their pathogenetic mechanisms, such as the upregulation of Th2 cytokines (IL-4 and IL-13), altered expression or loss-of-function mutations in atopy-associated genes (like filaggrin), and increased serum immunoglobulin E (IgE) levels. The Th1 response is the primary mediator in the pathogenesis of AA, but recent evidence has highlighted its role in the evolution of AD, particularly in the chronic phase. This overlap in cytokine profiles and genetic factors suggests that both conditions may benefit from therapies targeting these shared pathways, such as JAK inhibitors.

### 4.2. The Role of the JAK-STAT Pathway and Rationale for JAK Inhibitors

JAK1 is widely expressed in tissues and is responsible for mediating responses in both Th1 and Th2 pathways [24]. Notably, the JAK-STAT pathway is activated by numerous Th2 pro-inflammatory cytokines (IL-4, IL-13, IL-31, IL-22, TSLP), as well as by Th1 cytokines like IL-15 and IFN-γ [25]. Baricitinib, an oral inhibitor of JAK1 and JAK2, is the first systemic drug approved by the EMA in July 2022 for the treatment of severe AA in adult patients and the only one reimbursed in Italy from September 2023 [26]. Notably, the efficacy and safety of baricitinib in children and adolescents with AA have not yet been evaluated, and it is not approved in these patients. In September 2023, EMA approved Ritlecitinib, an oral inhibitor of JAK3 and tyrosine kinase expressed in the hepatocellular carcinoma family (TEC), for the treatment of severe AA in adults as well as in adolescents aged 12 years and older [27]. However, Ritlecitinib has not demonstrated efficacy for atopic AD.

### 4.3. Efficacy and Safety of Upadacitinib in Pediatric Population

Upadacitinib is a selective JAK1 inhibitor approved for several autoimmune diseases, including rheumatoid arthritis, psoriatic arthritis, ankylosing spondylitis, non-radiographic axial spondyloarthritis, ulcerative colitis, Crohn’s disease, and moderate-to-severe AD in adults and adolescents aged ≥12 years [28]. Its mechanism of action targets key pro-inflammatory cytokines involved in both Th1- and Th2-mediated pathways, such as IFN-γ, IL-4, IL-13, and IL-22, offering a rationale for its efficacy in complex immune-mediated conditions like AD and AA [29]. In adolescents with AD, upadacitinib has demonstrated a favorable safety and efficacy profile comparable to that observed in adults. Common adverse events include acne, upper respiratory tract infections, and mild creatine phosphokinase elevations, generally manageable without treatment discontinuation [30]. The recommended dosage for adolescents is 15 mg once daily [11].

### 4.4. Literature Review

The literature includes various case reports on the use of upadacitinib for the treatment of AA in adult patients [31]. To date, only four cases of concomitant AA and AD in pediatric patients treated with upadacitinib have been described. Kotcz K. et al. reported on a 14-year-old female patient affected by both AA (SALT score of 100) and AD. The patient had previously been treated with topical 5% minoxidil and mometasone furoate, immunotherapy with diphenylcyclopropenone (DCP), and narrowband UVB (NB UVB), with limited response. Her AD was well controlled with topical corticosteroids and calcineurin inhibitors. The patient started upadacitinib 15 mg with a complete response (SALT score of 0) after three months of treatment. The only reported side effect was transient mild leukopenia, which was resolved by the 12-week follow-up exam [19]. Bourkas A.N. et al. presented a case of a 14-year-old patient with AA previously treated with intralesional corticosteroid injections, high-potency topical corticosteroids, and calcineurin inhibitors cream for the scalp. The patient also had concurrent AD, managed with topical corticosteroids during flare-ups. After the initial visit, cyclosporine at 5 mg/kg/day was initiated and was well tolerated, with an initial good response in both AA and AD, followed by a relapse after 7 months. Low-dose systemic minoxidil yielded minimal response, and methotrexate was administrated and replaced with upadacitinib after one month (unknown dose). After 5 months, the patient achieved a complete response with a SALT score of 0 and marked improvement in eczema. No adverse events were reported [18]. Yu et al. reported a pediatric case of alopecia universalis (SALT score 98) in a 9-year-old patient concurrent with AD, unresponsive to treatment with potent topical steroids, minoxidil, tacrolimus, oral compound glycyrrhizin tablets, and glucocorticoids. The patient was treated with upadacitinib 15 mg, achieving complete clinical remission (SALT score 9) in five months without any significant adverse events [20]. Gi ung reported a case of a 15-year-old female patient presenting severe AA (SALT score 100) and a history of AD. She was previously treated with cyclosporine, steroid mini pulse and injections, DPCP, excimer laser, and cryotherapy with no improvement. Upadacitinib 15 mg was started, and after 12 months, a complete response was observed (SALT 11.7) with AD good control [21].

### 4.5. Our Findings

These case reports suggest the effectiveness of upadacitinib in treating pediatric patients with severe AA and concurrent AD, demonstrating significant improvements in both conditions. Complete responses were observed within several months of treatment, with minimal or no adverse events reported. However, further evidence is needed to support the efficacy of the drug in children, for which only one case is reported in the literature. Our study reports three cases of AA in adolescent patients effectively treated with upadacitinib. All our patients presented concurrent mild to moderate AD. Two patients (Cases 1 and 3) presented with a baseline SALT score of 100 and involvement of eyelashes and eyebrows (EBA and ELA of 0), while the third patient with ophiasis (Case 2) had a SALT score of 51.5. After just one month of therapy, all three patients showed some degree of improvement, albeit limited. After 6 months of treatment, substantial regrowth of eyelashes, eyebrows, and scalp hair was observed in Cases 1 (SALT 21.8 and EBA-ELA of 3) and 2 (SALT 15), with further improvement observed at the 12-month follow-up visit (SALT 14 and 1,8). The third patient, at the last six-month evaluation visit, achieved a SALT of 45 and EBA and ELA of 1. After an initially slower response, at the 12-month follow-up, she showed complete hair regrowth with a SALT score of 100 and partial regrowth of eyebrows and eyelashes (EBA and ELA of 2). Upadacitib outcome on AD was evaluated, with all patients showing good disease control after just one month of therapy. None of the three patients developed significant side effects. Our findings provide valuable evidence supporting the use of upadacitinib at a 15 mg dosage as a safe and effective treatment option for both adolescents and children with AA and AD. Notably, a slight clinical improvement in AA was observed after just one month of therapy, which, although modest, may predict the more substantial responses seen at 3, 6, and 12 months. We also present the first documented case of ophiasis successfully treated with upadacitinib, which typically has a poorer prognosis than other forms of AA and is often refractory to conventional treatments [32]. Additionally, we report two cases of successful upadacitinib use in pediatric patients with AA and AD following the failure of dupilumab therapy. This evidence underscores the potential of targeting the JAK-STAT pathway to manage complex conditions like AA and AD, which involve multiple immunological pathways. The third patient, who also had CD, reported an improvement in his gastrointestinal condition, which had previously been poorly controlled.

### 4.6. Limitations

Among the limitations, this is a retrospective study; however, all patients were evaluated during follow-up visits by an expert dermatologist specialized in trichology, both clinically and through dermoscopic examination. Moreover, the small sample size represents another limitation, likely due to the young age of the patients and parental concerns regarding the initiation of this novel JAK1 inhibitor therapy. Additionally, the current literature on the use of upadacitinib in pediatric patients is largely limited to case reports, which may introduce a positive selection bias. Nonetheless, the marked clinical improvements observed in our patients are encouraging and support further investigation.

## 5. Conclusions

Upadacitinib has demonstrated promising efficacy in the management of pediatric AD, leading to rapid and significant improvements in pruritus and sleep disturbances and achieving complete disease control as defined by MDA. No adverse effects were observed in our patient cohort. Furthermore, upadacitinib has shown effectiveness in patients with concomitant AD- and Th1-mediated comorbidities, such as AA and CD, with near-complete remission of both conditions reported within 6 to 12 months. We recommend trichoscopic evaluation at one month, as it may serve as an early marker of therapeutic response, with subsequent follow-ups at 3, 6, and 12 months. Nevertheless, additional studies are necessary to assess the long-term safety and efficacy of upadacitinib in larger pediatric populations. The management of AA and AD in children remains complex; however, the emergence of small-molecule therapies offers a valuable new avenue for improving patient outcomes.

## Figures and Tables

**Table 1:** Baseline characteristics of AA patients treated with upadacitinib.

Case Number	Age (Years)/Sex (M/F)	Atopic Comorbidities	AD Duration and Severity	Non-Atopic Comorbidities	AA Duration and Severity	Prior Treatment
1	13/M	AD, allergic rhinoconjunctivitis	Early onset; moderate EASI: 10 IGA: 1 Itch NRS: 6 Sleep NRS: 4 DLQI: 10	None	1 year; AU (SALT score 100)	Dupilumab
2	12/M	AD	Early onset; moderate EASI: 9 IGA: 1 Itch NRS: 6 Sleep NRS: 4 DLQI: 8	None	3 months; ophiasis (SALT score 51.5)	TSC, SCS, CsA
3	14/M	AD	Early onset; moderate EASI: 8 IGA: 2 Itch NRS: 6 Sleep NRS: 5 DLQI: 10	CD, EGE	17 months; AU (SALT score 100)	SCS, dupilumab

M: male; F: female; AD: atopic dermatitis; CD: Crohn’s disease; EGE: eosinophilic gastroenteritis; SALT: Severity of Alopecia Tool; AU: alopecia universalis; EASI: Eczema Area and Severity Index; IGA: Investigator Global Assessment; Itch NRS: Itch Numerical Rating Scale; Sleep NRS: Sleep Numerical Rating Scale; CsA: cyclosporine; SCS: systemic corticosteroids.

**Table 2:** Therapeutic regimen of upadacitinib and AA/AD scores during follow-up visits.

Case Number	Daily Dosage	Treatment Duration, Months	SALT Scores at Follow-Up Visits	EBA (0–3)	ELA (03)	AD Scores at 1-Month Follow-Up Visit	Adverse Events
1	15 mg	12	1 month: 96 3 months: 35.3 6 months: 21.8 12 months: 14	Baseline: 0 1 month: 1 3 months: 2 6 months: 3	Baseline: 0 1 month: 1 3 months: 2 6 months: 3	EASI: 0 IGA: 0 Itch NRS: 0 Sleep NRS: 0 CDLQI: 0	None
2	15 mg	12	1 month: 47.8 3 months: 35.3 6 months: 15 12 months: 1.8	NI	NI	EASI: 0 IGA: 0 Itch NRS: 0 Sleep NRS: 0 CDLQI: 0	Mild transient CPK elevation
3	15 mg	12	1 month: 92 3 months: 78 6 months: 45 12 months: 0	Baseline: 0 1 month: 0 6 months: 1 12 months: 2	Baseline: 0 1 month: 0 6 months: 1 12 months: 2	EASI: 0 IGA: 0 Itch NRS: 0 Sleep NRS: 0 CDLQI: 0	Mild transient CPK elevation

EASI: Eczema Area and Severity Index; IGA: Investigator Global Assessment; Itch NRS: Itch Numerical Rating Scale; Sleep NRS: Sleep Numerical Rating Scale; CDLQI: Children’s Dermatology Life Quality Index; SALT: Severity of Alopecia Tool; CPK: creatine phosphokinase; EBA: Eyebrow Assessment; ELA: Eyelash Assessment; NI: Not Involved.

**Table 3:** Laboratory tests of the three patients treated with upadacitinib 15 mg at T0 (before the start of upadacitinib 15 mg), T1 (1 month after the start of upadacitinib 15 mg), T2 (3 months after the start of upadacitinib 15 mg), T3 (6 months after the start of upadacitinib 15 mg), and T4 (12 months after the start of upadacitinib 15 mg).

CASE 1	T0	T1	T2	T3	T4
Hb	13.7 g/dL	14.1 g/dL	14.0 g/dL	14.2 g/dL	14.1 g/dL
Neutrophils	2.8 × 10^3^/uL	3.4 × 10^3^/uL	3.2 × 10^3^/uL	3.0 × 10^3^/uL	3.2 × 10^3^/uL
Eosinophils	0.1 × 10^3^/uL	0.1 × 10^3^/uL	0.1 × 10^3^/uL	0.1 × 10^3^/uL	0.1 × 10^3^/uL
IgE	**1264.0 IU/mL**	**1150 IU/mL**	**364 IU/mL**	**252 IU/mL**	**256 IU/mL**
ESR	4 mm/h	4 mm/h	3 mm/h	3 mm/h	3 mm/h
CRP	0.6 mg/dL	0.6 mg/dL	0.5 mg/dL	0.5 mg/dL	0.4 mg/dL
Total Cholesterol	145 mg/dL	157 mg/dL	150 mg/dL	142 mg/dL	155 mg/dL
Triglycerides	46 mg/dL	79 mg/dL	62 mg/dL	71 mg/dL	57 mg/dL
AST	22 U/L	19 U/L	20 U/L	21 U/L	19 U/L
ALT	17 U/L	19 U/L	19 U/L	18 U/L	18 U/L
CPK	190 U/L	148 U/L	142 U/L	145 U/L	153 U/L
**CASE 2**					
Hb	14.0 g/dL	14.5 g/dL	14.1 g/dL	14.7 g/dL	14.3 g/dL
Neutrophils	1.96 × 10^3^/uL	4.18 × 10^3^/uL	1.98 × 10^3^/uL	2.23 × 10^3^/uL	2.15 × 10^3^/uL
Eosinophils	0.13 × 10^3^/uL	0.12 × 10^3^/uL	0.14 × 10^3^/uL	0.12 × 10^3^/uL	0.12 × 10^3^/uL
IgE	120 IU/mL	110 IU/mL	82 IU/mL	63 IU/mL	58 IU/mL
ESR	21 mm/h	19 mm/h	15 mm/h	11 mm/h	10 mm/h
CRP	3.22 mg/dL	1.42 mg/dL	0.8 mg/dL	0.7 mg/dL	0.3 mg/dL
Total Cholesterol	155 mg/dL	143 mg/dL	155 mg/dL	145 mg/dL	148 mg/dL
Triglycerides	105 mg/dL	110 mg/dL	66 mg/dL	69 mg/dL	72 mg/dL
AST	27 U/L	23 U/L	38 U/L	21 U/L	22 U/L
ALT	17 U/L	13 U/L	21 U/L	13 U/L	15 U/L
CPK	83.80 U/L	175 U/L	**278 U/L**	158 U/L	143 U/L
**CASE 3**					
Hb	16.2 g/dL	15.8 g/dL	15.6 g/dL	15.5 g/dL	15.4 g/dL
Neutrophils	4.14 × 10^3^/uL	3.88 × 10^3^/uL	3.37 × 10^3^/uL	3.57 × 10^3^/uL	3.83 × 10^3^/uL
Eosinophils	**1.13 × 10 ^3^ /uL**	0.85 × 10^3^/uL	0.76 × 10^3^/uL	0.75 × 10^3^/uL	0.35 × 10^3^/uL
IgE	40.2 IU/mL	39.9 IU/mL	38.2 IU/mL	38.1 IU/mL	38.0 IU/mL
ESR	9 mm/h	8 mm/h	5 mm/h	5 mm/h	4 IU/mL
CRP	0.3 mg/dL	0.3 mg/dL	0.2 mg/dL	0.2 mg/dL	0.04 mg/dL
Total Cholesterol	159.7 mg/dL	148 mg/dL	152.3 mg/dL	154.2 mg/dL	161 mg/dL
Triglycerides	54.9 mg/dL	24 mg/dL	31 mg/dL	36 mg/dL	35 mg/dL
AST	16 U/L	21 U/L	28 U/L	23 U/L	22 U/L
ALT	12 U/L	15 U/L	23 U/L	16 U/L	23 U/L
CPK	112 U/L	**382 U/L**	135 U/L	143 U/L	116 U/L

Hb: hemoglobin; IgE: immunoglobulin E; ESR: Erythrocyte Sedimentation Rate; CRP: C-Reactive Protein; AST: Aspartate Aminotransferase; ALT: Alanine Aminotransferase; CPK: creatine phosphokinase.

**Table 4:** Summary of previous studies on the efficacy of upadacitinib in treating pediatric/adolescent patients with alopecia areata.

Study	Age (Years)/Sex (M/F)	Atopic Comorbidities	AD Duration and Severity	Non-Atopic Comorbidities	AA Duration and Severity	Prior Treatment	Daily Dosage	Treatment Duration, Months	Treatment Outcome AA; AD	Side Effects
Bourkas et al. [18]	14/M	AD, allergic rhinitis, asthma, allergies to sesame seeds, grass, tree nuts, eggs	NA; severe	None	13 years; almost the entire scalp	ICSI, TCS, TCI, CsA, LDOM, MTX	NA	5	Complete response; marked improvement	NA
Kołcz et al. [19]	14/F	AD	Early childhood; mild	None	16 months; AU (SALT score 100)	TSC, TCI, TMX, DPCP, NB UVB	15 mg	3	Complete response (SALT score 0); complete response	Mild transient leukopenia
Yu et al. [20]	9/F	AD	NA; mild (EASI score 2.5)	None	7 years; AU (SALT score 98)	TCS, TMX, TCI, SCS, oral compound glycyrrhizin tablets	15 mg	5	Complete response (SALT score 0); complete remission	None
Ha et al. [21]	15/F	AD	NA; NA	None	11 years; AT (SALT score 100)	CsA, SCS, ICSI, DPCP, EL, CT	15 mg	12	SALT score 11.7; NA	None

M: male; F: female; AD: atopic dermatitis; NA: not available; EASI: Eczema Area and Severity Index; SALT: Severity of Alopecia Tool; AU: alopecia universalis; AT: alopecia totalis; ICSI: intralesional corticosteroids; TCS: topical corticosteroids; TCI: topical calcineurin inhibitors; CsA: cyclosporine; LDOM: low-dose oral minoxidil; MTX: methotrexate; DPCP: diphenylcyclopropenone; NB UVB: narrow band ultraviolet B; TMX: topical minoxidil; SCS; systemic corticosteroids; EL: excimer laser; CT: cryotherapy.

## Data Availability

The data that support the findings of this study are available from the corresponding author upon reasonable request.

## Supplementary Materials

The following supporting information can be downloaded at: https://www.mdpi.com/article/10.3390/jcm14113881/s1, Table S1: Clinician- and Patient-Reported Outcome Measures, Description, and Response Scale/Scoring. References [33,34,35,36,37] are cited in the Supplementary Materials.
