# Supplementary material for: Efficacy of Upadacitinib in Treating Alopecia Areata, Atopic Dermatitis, and Th1 Comorbidities in Pediatric Patients: A Comprehensive Case Series and Literature Review
Source: J Clin Med. 2025 May 30;14(11):3881. doi: 10.3390/jcm14113881 (PMC12155964; doi:10.3390/jcm14113881)
Supplement: Supplementary file 1 [file jcm-14-03881-s001.zip › jcm-3644228-supplementary.pdf]

Supplementary Table S1. Clinician- and Patient-Reported Outcome Measures, Description, and Response Scale/Scoring

Outcome Measure	Response Scale/Scoring/Questionnaire
Severity of Alopecia Tool (SALT)[1]<br>▪ Clinician assessment of the amount of scalp hair loss	▪ 0 (no scalp hair loss) to 100 (complete scalp hair loss)
Eyebrow Assessment (EBA)[2]<br>▪ Clinician assessment of the eyebrow hair loss	▪ 0 = **None** (no eyebrow hair)<br>▪ 1 = **Minima**l eyebrow (normal or decreased density of 1 or both eyebrows with large gap[s] or severely decreased density of 1 or both eyebrows with or without gap[s])<br>▪ 2 = **Moderate** eyebrow (normal density of both eyebrows with short gap[s] that does not significantly distort the appearance of the eyebrows or mildly decreased density of eyebrows with or without short gap[s] or moderately decreased density of eyebrows without short gaps[s]. There is a visual definition of eyebrows at a distance of 3 feet)<br>▪ 3 = **Normal** eyebrow (normal density of both right and left eyebrows spanning usual length (ie, from glabella to near temple) and width. There are no gaps)
Eyelash Assessment (ELA)[2]<br>▪ Clinician assessment of the eyelash hair loss	▪ 0 = **None** (no eyelash hair)<br>▪ 1 = **Minima**l eyelash (modestly or severely decreased density of and/or large gap[s] in 1 or both upper eyelashes)<br>▪ 2 = **Moderate** eyelash (normal density of both upper eyelashes without gap[s] and decreased density or gap[s] is present in one or both lower eyelashes or normal density of both upper eyelashes with short gap[s] or mildly decreased density of one or both upper eyelashes with or without short gap[s])<br>▪ 3 = **Normal** eyelash (normal density of both right and left upper and lower eyelashes from near medial canthus to near lateral canthus without any gap[s])
Eczema Area and Severity Index (EASI)[3]<br>▪ Clinician assessment of the severity and extent of AD	Four AD disease characteristics (erythema, thickness [induration, papulation, edema], scratching [excoriation], and lichenification) are assessed for severity on a scale of ‘0’ (absent), ‘1’ (mild), ‘2’ (moderate), and ‘3’

	(severe). The area of AD involvement are assessed as a percentage by body area of head, trunk, upper limbs, and lower limbs, and converted to a score of 0 to 6. In each body region, the area is expressed as 0, 1 (1% to 9%), 2 (10% to 29%), 3 (30% to 49%), 4 (50% to 69%), 5 (70% to 89%), or 6 (90% to 100%).<br><br>For each region, the severity of the four AD signs—redness, thickness, scratching, and lichenification—are recorded and used to calculate the severity score.<br><br>The severity score is calculated as follows: Severity score = redness severity + thickness severity + scratching severity + lichenification severity.<br><br>For each region, the severity is multiplied by the area score and by a region-specific multiplier as outlined below:<br><br>• Head and neck: severity score x area score x 0.1<br>• Trunk: severity score x area score x 0.3<br>• Upper limbs: severity score x area score x 0.2<br>• Lower limbs: severity score x area score x 0.4<br><br>The total scores for each region are then summed to determine the final EASI score, which ranges from 0 to 72.
Investigator's Global Assessment (IGA)[3]<br>▪ Clinician assessment of the global severity of AD	▪ 0 = **Clear** (no inflammatory signs of AD [no erythema, no induration/papulation, no lichenification, no oozing/crusting]. Post-inflammatory hyperpigmentation and/or hypopigmentation may be present.)<br>▪ 1 = **Almost clear** (barely perceptible erythema, barely perceptible induration/papulation, and/or minimal lichenification. No oozing or crusting.)<br>▪ 2 = **Mild** (slight but definite erythema [pink], slight but definite induration/papulation, and/or slight but definite lichenification. No oozing or crusting.)

	▪ 3 = **Moderate** (clearly perceptible erythema [dull red], clearly perceptible induration/papulation, and/or clearly perceptible lichenification. Oozing and crusting may be present.)<br>▪ 4 = **Severe** (marked erythema [deep or bright red], marked induration/papulation, and/or marked lichenification. Disease is widespread in extent. Oozing and crusting may be present.)
Itch Numerical Rating Scale (Itch NRS)[4]<br>▪ Patient assessment of the intensity of their pruritus (itch) during a daily recall period	'On a scale of 0-10, how would you rate your itch overall (on average) during the previous 24 hours?'<br>▪ 0 (no itch) to 10 (worst itch imaginable)
Sleep Numerical Rating Scale (Sleep NRS)[4]<br>▪ Patients assessment of the sleep quality last night	'On a scale of 0-10, how would you rate your sleep last night?'<br>▪ 0 (no sleep loss related to the symptoms of AD) to 10 (not sleep at all due to the symptoms of AD)
Children's Dermatology Life Quality Index (CDLQI)[5]<br>▪ Patient assessment of the impact of AD disease symptoms and treatment on QOL	1. Over the last week, how **itchy, "scratchy", sore** or **painful** your skin been?<br>2. Over the last week, how **embarrassed** or **self conscious, upset** or **sad** have you been because of your skin?<br>3. Over the last week, how much has your skin affected your **friendships**?<br>4. Over the last week, how much have you changed or worn **different** or **special clothes/shoes** because of your skin?<br>5. Over the last week, how much has your skin trouble affected **going out, playing, or doing hobbies**?<br>6. Over the last week, how much have you avoided **swimming** or **other sports** because of your skin trouble?<br>7. Over the last week, was it **school time OR** was it **holiday time**? If **school time**: Over the last week, how much did your skin problem affect your **school work**? If **holiday time**: How much over the last week, has your skin problem interfered with your enjoyment of the **holiday**?<br>8. Over the last week, how much trouble have you had because of your skin with other people **calling you names, teasing, bullying, asking question** or **avoiding you**?<br>9. Over the last week, how much has your **sleep** been affected by your skin problem?

	10. Over the last week, how much of a problem has the **treatment** for your skin been?<br><br>For each question, the possible answers with their respective scores are: very much (3), quite a lot (2), only a little (1), not at all (0)<br><br>▪ 0 (no effect at all on patient's life) to 30 (extremely large effect on patient's life)

AD: Atopic Dermatitis; QOL: Quality Of Life

References:

1) Olsen EA, Hordinsky MK, Price VH, et al. Alopecia areata investigational assessment guidelines—Part II. *J Am Acad Dermatol.* 2004 Mar;51(3):440–447. Doi:10.1016/j.jaad.2003.08.013
2) King B, Guttman-Yassky E, Peeva E, et al. A phase 2 trial of baricitinib for alopecia areata. *N Engl J Med.* 2021;384(18):1687–1699. Doi: 10.1056/NEJMoa2026320
3) Hanifin JM, Thurston M, Omoto M, Cherill R, Tofte SJ, Graeber M. The eczema area and severity index (EASI): assessment of reliability in atopic dermatitis. EASI Evaluator Group. Exp Dermatol. 2001 Feb;10(1):11-8. Doi: 10.1034/j.1600-0625.2001.100102.x.
4) Yosipovitch G, Reaney M, Mastey V, et al. Validation of the Worst Itch Scale (WIS) and Sleep NRS in patients with moderate-to-severe atopic dermatitis. *J Am Acad Dermatol.* 2019;80(6):1452–1459. Doi: 10.1016/j.jaad.2018.11.057
5) Lewis-Jones MS, Finlay AY. The Children's Dermatology Life Quality Index (CDLQI): initial validation and practical use. Br J Dermatol. 1995 Jun;132(6):942-9. Doi: 10.1111/j.1365-2133.1995.tb16953.x.
